# Survival Outcomes in Invasive Lobular Carcinoma Compared to Oestrogen Receptor-Positive Invasive Ductal Carcinoma

**DOI:** 10.3390/cancers13123036

**Published:** 2021-06-18

**Authors:** Jasmine Timbres, Charlotte Moss, Anca Mera, Anna Haire, Cheryl Gillett, Mieke Van Hemelrijck, Elinor Sawyer

**Affiliations:** 1 Breast Cancer Genetics, King’s College London, London SE1 9RT, UK; elinor.sawyer@kcl.ac.uk; 2 Translational Oncology and Urology Research, King’s College London, London SE1 9RT, UK; charlotte.moss@kcl.ac.uk (C.M.); anna.haire@kcl.ac.uk (A.H.); mieke.vanhemelrijck@kcl.ac.uk (M.V.H.); 3 Guy’s & St. Thomas’ Hospital, London SE1 9RT, UK; anca.mera@kcl.ac.uk; 4 KHP Cancer Biobank, King’s College London, London SE1 9RT, UK; cheryl.gillett@kcl.ac.uk

**Keywords:** breast cancer, lobular, ductal, chemotherapy, survival, cohort study, retrospective

## Abstract

**Simple Summary:**

Around 10–15% of breast cancer diagnoses are invasive lobular cancers (ILC), and they are currently treated in a similar way to the more common invasive ductal cancer (IDC), although they display different characteristics. The main objective of this study was to identify any differences in outcome following chemotherapy treatment between ILC and oestrogen receptor-positive (ER+) and human epidermal growth factor receptor 2-negative (HER2−) IDC. Results from the analysis show worse survival in patients with ER+HER2− ILC after chemotherapy compared to ER+HER2− IDC, even after correcting for tumour size, grade, age, and nodal involvement at presentation, suggesting a worse response to chemotherapy in ILC. Thus, recommendations for chemotherapy treatment should be considered separately for the two subtypes. However, this association should be studied in a larger population to confirm this finding.

**Abstract:**

Invasive lobular breast cancer (ILC) accounts for 10–15% of breast cancers and has distinct characteristics compared with the more common invasive ductal carcinoma (IDC). Studies have shown that ILC may be less sensitive to chemotherapy than IDC, with lower rates of complete pathological response after neo-adjuvant chemotherapy, but it is not clear how this affects long-term survival. Patients at Guy’s and St Thomas’ NHS Foundation Trust between 1975 and 2016 diagnosed with ER+ IDC or ER+ ILC were eligible for inclusion. Kaplan–Meier plots and Cox proportional-hazards regression models were used for analysis. There was no difference in overall survival comparing ER+ ILC to ER+ IDC (OR: 0.94, 95% CI: 0.83, 1.04) with a median follow-up time of 8.3 years compared to 8.4 years in IDC. However, ER+HER2− ILC had worse survival compared to ER+HER2− IDC in those that received chemotherapy (OR: 1.46, 95% CI: 1.06, 2.01). Here, median follow-up time was 7.0 years in ILC compared to 8.1 years in IDC. These results indicate worse overall survival after chemotherapy (neo-adjuvant and adjuvant) in ILC compared to ER+HER2− IDC even when correcting for tumour grade, age, size, and nodal involvement, but validation is needed in a larger study population.

## 1. Introduction

Breast cancer is a heterogenous disease with various histological subtypes, each of which have different characteristics [1]. Invasive lobular cancer (ILC) is the second most common breast cancer subtype after invasive ductal carcinoma (IDC), accounting for 10–15% of all newly diagnosed breast malignancies, and is characterised by a lack of E-cadherin expression, and small, round, discohesive cells which grow in the stroma in single-file fashion, making them difficult to detect clinically, and mammographically, often presenting as architectural distortion [1,2,3,4].

Clinicopathological features in ILC differ to IDC, with most being grade 2 and expressing oestrogen (ER+) and progesterone (PR+) receptors. A minority of ILC overexpress the human epidermal growth factor receptor 2 (HER2), which is mainly attributed to the pleomorphic subtype [5], and this overexpression was associated with a worse prognosis in breast cancer [2,6], until the advent of anti-HER2 therapies. The published literature has demonstrated that ILC often presents at a later disease stage compared to IDC [7]. ILC is also more likely to be multifocal than IDC [8], and is more likely to need re-excision of margins after breast-conserving surgery due to positive margins [9].

Reports have not been consistent regarding differences in outcomes after IDC and ILC [7,10,11,12], with some evidence of better short-term survival in ILC than IDC, but worse outcomes in ILC than IDC after 10 years of follow up, even when restricted to ER+ ILC and ER+ IDC [11]. This is despite ILC being more likely to display characteristics that would indicate a better prognosis, for example being hormone receptor positive, and lacking HER2 over-expression (HER2−) [13,14]. Nevertheless, treatment guidelines for breast carcinoma continue to be informed primarily by the conclusions of clinical research studies involving the IDC subtype specifically, and do not account for the distinct molecular and clinicopathological features of ILC. Furthermore, current guidelines for prescribing chemotherapy do not take into account morphological subtype, despite emerging literature observing that ILC may be less chemosensitive than IDC [15,16] with less complete pathological responses (pCR) following neo-adjuvant chemotherapy (NACT) [17,18]. This is because it is not clear whether this lack of chemosensitivity also affects survival.

Data on outcomes after adjuvant chemotherapy (ACT) is also limited. Truin [19] did compare ER+ ILC to IDC, and observed no considerable difference in survival after treatment with both adjuvant endocrine therapy and ACT between the two histological subtypes. Several other studies have shown that ACT did not improve survival for patients with hormone receptor (HR)-positive, early-stage ILC, when compared to ILC treated with endocrine therapy alone [16,20]. However, these studies did not directly compare overall survival to ER+ IDC, and so it is not clear whether this finding is confined to just ILC, or if it also applies to ER+ IDC of similar stage. The aim of the current study is to evaluate survival after chemotherapy in ILC, by directly comparing overall survival in patients with ER+HER2− ILC to ER+HER2− IDC, diagnosed at Guy’s and St Thomas’ Hospital (GSTT) between 1975 and 2016.

## 2. Materials and Methods

### 2.1. Participants

Patients for this retrospective cohort study were chosen from both the King’s Health Partners Breast Cancer Biobank and the Breast Cancer Clinical Database at Guy’s and St Thomas’ Hospital NHS Foundation Trust (GSTT) in London, UK. Patients were considered for inclusion if pathology samples from the KHP Cancer Biobank showed that they had been diagnosed with either IDC or ILC. The use of data from the Biobank was permitted in this study under the approval of the Guy’s NHS Research Ethics Committee for patients diagnosed up to September 2006 (REC Number: 12/EE/0493) and individual consent was obtained from patients diagnosed after this date. The use of data from the Breast Cancer Clinical Database was permitted under Guy’s Cancer Cohort (REC 18/NW/0297). The study sample size was determined to be the number of available patients according to the inclusion criteria.

For the purposes of this study, all consenting female patients who received a new diagnosis of IDC or ILC between 01/01/75 and 31/12/2016 were selected for inclusion. Only patients with invasive ductal carcinoma and invasive lobular carcinoma were included; those with mixed invasive disease or other histological subtypes were excluded from the study population. In addition, any patients with IDC or ILC who had oestrogen receptor-negative disease, or for whom oestrogen receptor status data was missing, were excluded. Patients with multifocal or bilateral tumours of different molecular subtypes were also excluded from analysis. The final study cohort was comprised of 4276 ER+ IDC patients and 633 patients diagnosed with ER+ ILC.

In the patients who received chemotherapy as treatment for their primary breast cancer, all patients with HER2-positive tumours were excluded from analysis, as were those for whom the HER2 status was missing. This decision was made as ILC tends to be ER+ and HER2−, and this would allow for a comparison group which would have received similar treatments.

### 2.2. Data

The GSTT Breast Cancer Clinical Database contains prospectively collected data obtained from medical records and hospital systems for patients diagnosed with breast cancer at the NHS Trust. The King’s Health Partners Breast Cancer Biobank contains tissue and blood samples alongside histopathological data on such samples for patients diagnosed with breast cancer at the NHS Trust. Data covering patient demographics, tumour characteristics, and treatment were extracted from the clinical database for the purposes of this study. The age of patients at diagnosis was calculated using recorded date of birth and the date of histological diagnosis of breast cancer, and was further simplified into a binary variable, for below/above 50 years of age. Family history was self-reported and, following the consensus that any family history is associated with an increased risk of breast cancer, patients with third-degree family history were grouped separately from those with no family history. Menstrual status was simplified into pre- and post-menopausal categories, with peri-menopausal and lactating/pregnant forming part of the pre-menopausal grouping.

Nodal status, tumour size, and the presence of distant and local spread of disease were reported from pathology records, and anatomic staging was calculated using these variables according to the American Joint Committee on Cancer (AJCC) 7th Edition Cancer Staging Manual [21]. Invasive size of the tumour was reported pathologically for most patients but, in cases where such data was missing, was estimated from data collected during clinical examination. Invasive grade was also reported from histopathological records using the Nottingham combined histologic grading system (grade 1, grade 2, grade 3), as were all other tumour variables, such as ER/PR/HER2 status. Although the majority of ILC are moderately differentiated and reported as grade 2 at diagnosis, at the time of study baseline the prognostic value of grading ILCs had not been fully elucidated and therefore missing data were an issue in the ILC subset of patients [22,23]. For the purposes of this study, any missing data on grade within the ILC subset of the study population were assumed to be grade 2. Oestrogen receptor status was inferred as positive in cases where treatment data indicated that hormonal therapies were given.

Clinical follow-up information for all patients was collected as per hospital protocol until 1 October 2020. Patients who had not been re-referred to the clinic by 1 October 2020, and for whom there was no date of death reported, were assumed to be alive and well with no local or distant recurrence. All follow-up information was extracted from hospital medical records and included treatment regimens, date of disease recurrence, and date of last contact, with date of death determined from death certificates or mortality reports. Information on death was obtained from the National Cancer Registration and Analysis Service (Public Health England), the Office of National Statistics, and Summary Care Records, with cause of death confirmed by National Death Certificate data.

### 2.3. Analysis

Comparisons of characteristics were performed using unpaired t-tests for continuous variables, or either Pearson’s χ^2^ or Fisher’s exact tests for categorical variables. Overall survival (OS) time was defined as the time between diagnosis to death of any cause, or end of study follow-up (1 October 2020) if no death data was available. Recurrence-free survival (RFS) time was the time between diagnosis and either local or distant recurrence of disease, while metastasis-free survival (MFS) referred to the time between diagnosis and distant recurrence of disease only.

Survival analyses based on histological type of breast carcinoma were conducted using the Kaplan–Meier product limit method, and log rank tests of equality of survivor functions were used to compare differences between the two main histological groups. The association between the histological subtypes of breast carcinoma and overall survival was further analysed using multivariate Cox proportional-hazards (PH) regression models. In those who received chemotherapy, analysis was not stratified by chemotherapy type in order to explore the effect of any type of chemotherapy on survival, and because numbers in the NACT group were small. The proportional-hazards assumption for all Cox proportional-hazards models were evaluated by visual inspection of log–log plots. Further to this, landmark analyses were undertaken at 5 years, where those with less than 5 years of survival after their breast cancer diagnosis were excluded. This allowed for the evaluation of the association considering well-described changes in survival in ILC compared to IDC.

Statistical analyses were performed using Statistical Analysis Systems (SAS) release 9.3 (SAS Institute, Cary, NC, USA) and STATA/MP 16.0.

## 3. Results

**Figure 1:**
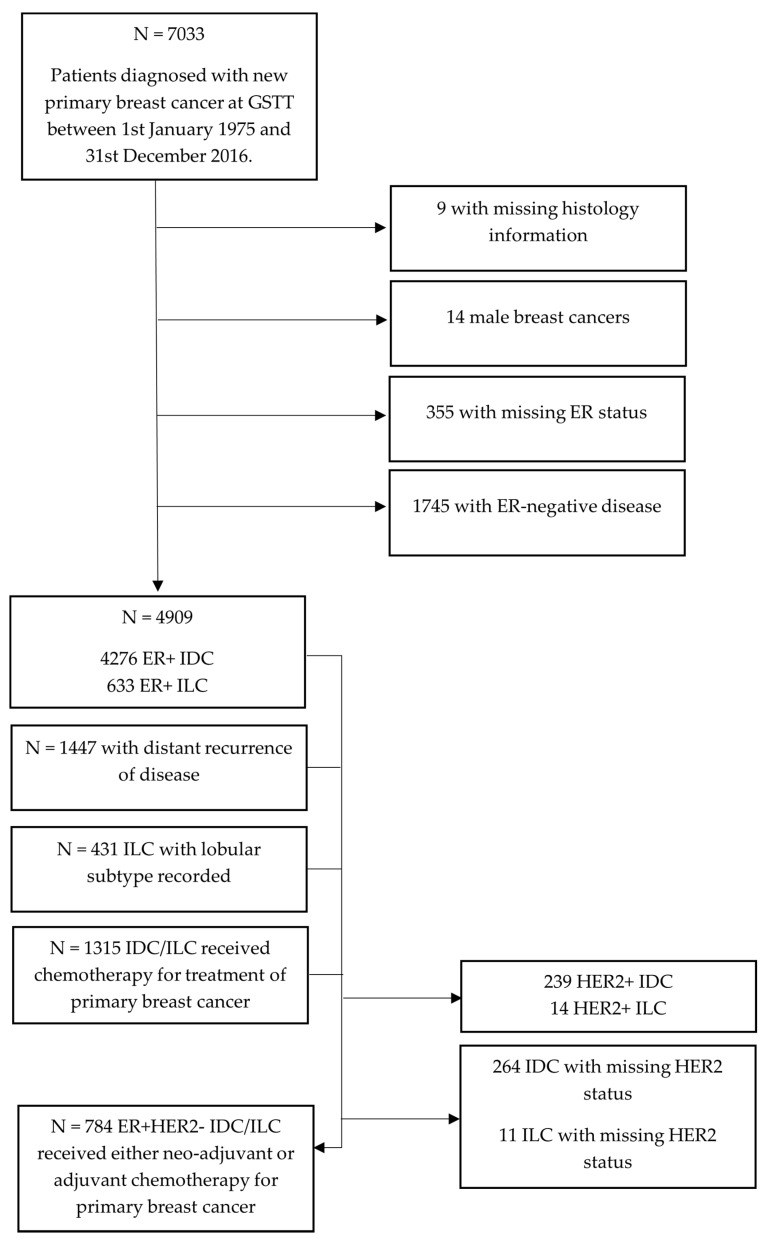
Flow chart of patients included in the study from the GSTT Breast Cancer Database.

Of 7033 patients diagnosed with either ILC or IDC between 1975 and 2016, 4909 ER+ patients were analysed as part of this study, of which 4276 (87.1%) had a diagnosis of ER+ IDC, and 633 (12.9%) had a diagnosis of ER+ ILC (Figure 1). Of these 4909 ER+ patients, 1315 received chemotherapy as part of their primary cancer treatment, but after exclusions of HER2+ and HER2-missing cases, 784 ER+ patients were included for analysis of survival after chemotherapy. Of the 4909 ER+ patients, 1447 developed a distant recurrence.

Women with ER+ ILC tended to be older (median age ER+ IDC 57.2 years, ER+ ILC 59.6 years, *p* ≤ 0.001), and diagnosed in a greater proportion of women above the age of 50 (*p* ≤ 0.001), although this association was not as significant in patients treated with chemotherapy (*p* = 0.045) (Table 1). Follow-up time was slightly lower for ILC, with a median of 8.3 years (range: 0.1–44.2 years), in comparison to IDC with a median follow-up of 8.4 years (range: 0–45.7 years). No significant difference in number of births was observed when comparing ER+ IDC and ER+ ILC, which is in line with previous studies showing that parity is protective against both subtypes similarly [24,25,26,27], although there was a high proportion of missing data on births in this study. However, there have been conflicting results, with some studies observing that increasing parity decreases the risk of IDC, but not ILC [28,29,30,31]. There were also no obvious differences between IDC and ILC in regards to family history or ethnicity, although data was not complete for these variables. Over the course of 1975–2016, the diagnosis of both lobular and ductal breast cancer increased overall (Figure S1), although only half of the first and last decades were included in this study. These increases in ILC could be attributed to the increased uptake of hormone replacement therapies, as previous studies have reported stronger associations between hormone replacement therapies and ILC than IDC [24]. However, it is also possible that increases in incidence over time are related to lifestyle risk factors such as diet and obesity, or changes in reproductive behaviours.

As expected, differences in tumour grade, tumour size, and nodal involvement were seen between IDC and ILC (Table 2), with a higher proportion of T2 and T3 tumours in ILC than IDC. The proportion of IDC and ILC patients receiving NACT/ACT was similar (27% for IDC, 25% for ILC), but within the cases that received chemotherapy, a higher percentage of ILC were locally advanced (T4) tumours. In the group that received chemotherapy, there were less node-negative disease cases compared to the wider ER+ group, and a higher proportion with extensive nodal spread (N2/N3).

### 3.1. Survival after ER+ Breast Cancer

**Figure 2:**
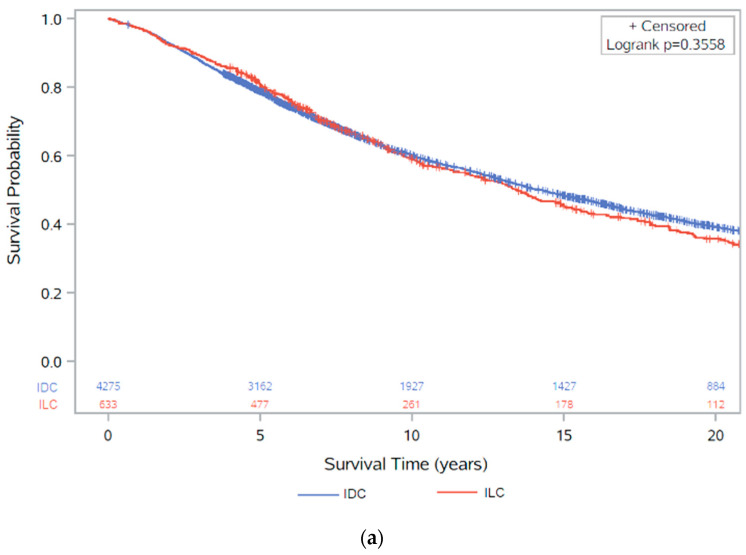

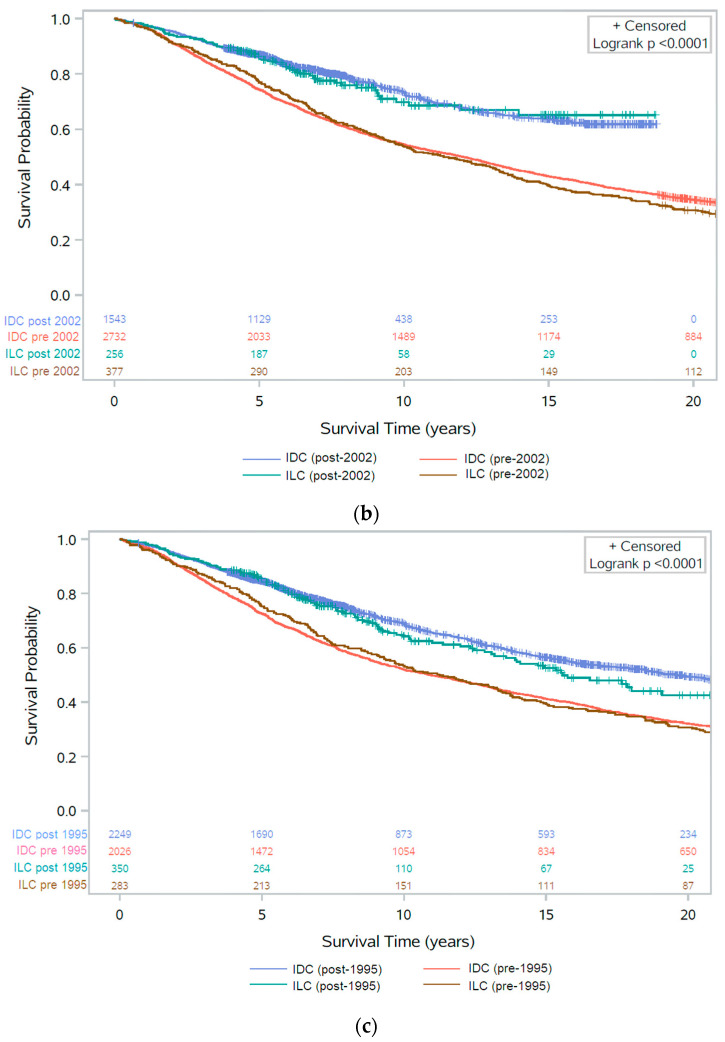
Kaplan–Meier survival analysis of overall survival (with number of subjects at risk): (**a**) between ER+ IDC and ILC (*p* = 0.3558); (**b**) between ER+ IDC and ILC, stratified by diagnosis pre/post 2002 (*p* < 0.0001); (**c**) between ER+ IDC and ILC, stratified by diagnosis pre/post 1995 (*p* < 0.0001). These figures show results for patients with ER+ disease, irrespective of HER2 status.

Overall survival was compared for ER+ IDC and ER+ ILC, as shown by the Kaplan–Meier curves in Figure 2. Over the follow-up period, 2831 (57.7%) patients died, of which, 1355 (47.8%) deaths were from breast cancer; however, data on cause of death was missing for ~40% of patients. Survival was slightly better in ER+ ILC than ER+ IDC at 5 years after diagnosis, although after 10-years of follow-up, there was no clear difference in survival, and in the longer term, survival was lower in patients with ILC (Figure 2a).

As Herceptin was only approved for use in metastatic breast cancer in the UK in 2002, and for early-stage cancers in 2006 [32,33], survival was also stratified into diagnosis before or after 2002 to investigate the effect of the introduction of Herceptin as a treatment for breast cancer (Figure 2b). Here, the initial survival benefit of ER+ ILC was only seen in cases diagnosed before 2002. In those diagnosed after 2002, there was little difference in survival between ER+ IDC and ER+ ILC, although ILC saw worse survival between 5 and 10 years. Survival was also stratified into diagnosis before or after 1995, accounting for treatment changes over time such as the use of anthracycline-based chemotherapy [34] (Figure 2c). Here, survival was worse before 1995 compared to after 1995, showing that improvement in treatment have had an effect on survival, but this is more pronounced in IDC.

The univariate Cox regression model found no clear difference in overall survival between ER+ ILC and ER+ IDC (Table 3). The multivariate Cox proportional-hazards models including age at diagnosis, invasive grade, tumour size, nodal involvement, HER2 status, and chemotherapy treatment also showed no evidence of a difference in survival, with a hazard ratio of 0.94 (95% CI: 0.83, 1.04) for overall survival in patients with ER+ ILC compared to ER+ IDC. Cox proportional-hazards models were also stratified by length of follow-up due to changes in survival between IDC and ILC observed in the Kaplan–Meier survival curve. The proportional-hazards assumption was evaluated by visual inspection of a log–log plot (Figure S2), and the assumption was assumed to be valid as the lines for each group did not meet or cross, indicating proportional hazards.

Similarly, for the 3639 ER+ cases that had at least 5 years of survival time, the univariate Cox regression analysis showed a borderline significant estimate of 15% worse survival in ER+ ILC compared to ER+ IDC (95% CI: 1.00, 1.32), but this association was no longer significant when adjusted by nodal status, tumour size, grade, age at diagnosis, HER2 status, and chemotherapy (OR: 0.97, 95% CI: 0.83, 1.12) (Table S1).

### 3.2. Survival after ER+ Breast Cancer Treated with Chemotherapy

**Figure 3:**
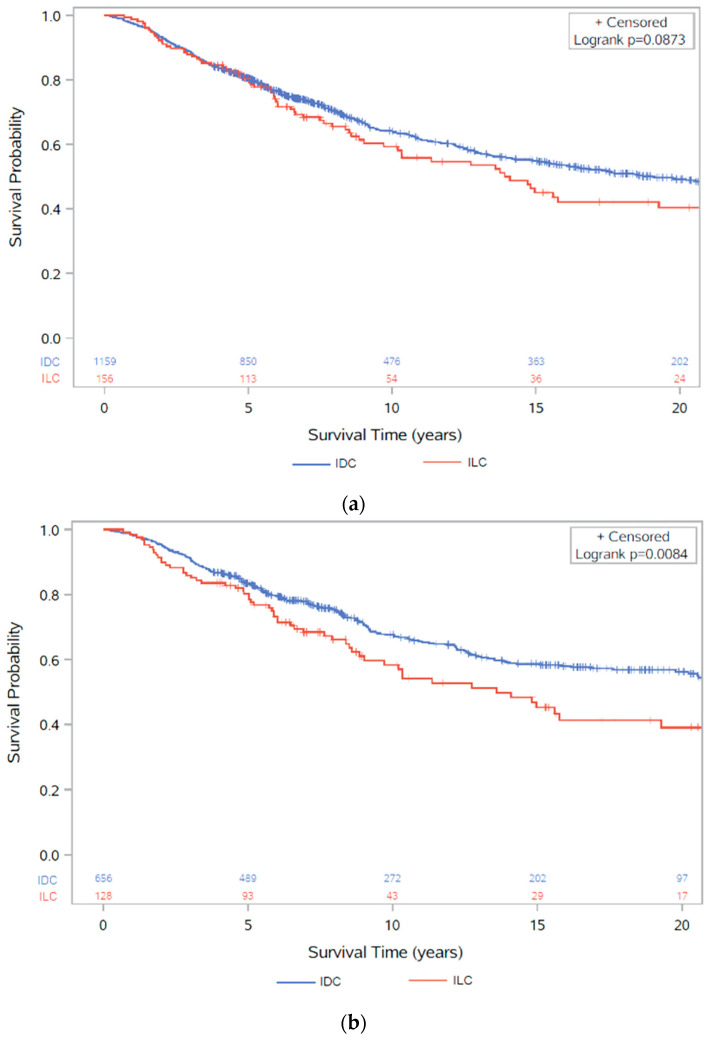

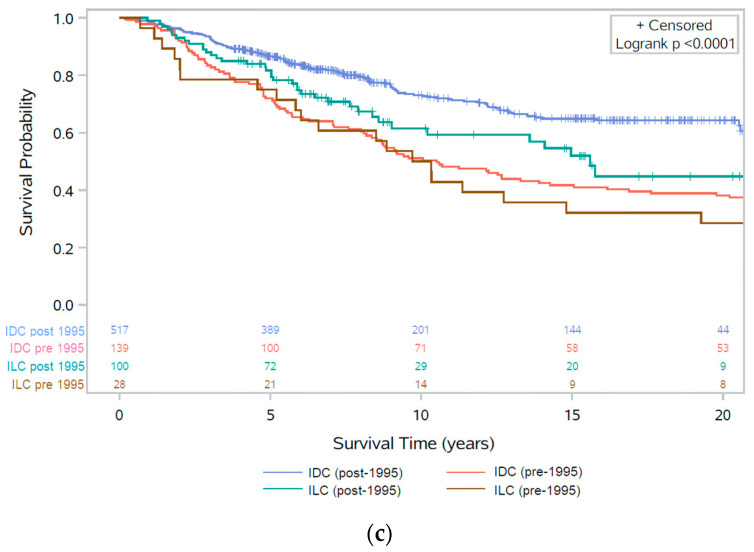
Kaplan–Meier survival analysis of overall survival (with number of subjects at risk): (**a**) between ER+ IDC and ER+ ILC after chemotherapy (*p* = 0.0873) irrespective of HER2 status; (**b**) between ER+HER2− IDC and ER+HER2− ILC after chemotherapy (*p* = 0.0084); (**c**) between ER+HER2− IDC and ER+HER2− ILC after chemotherapy before and after 1995 (*p* < 0.0001).

In 1315 ER+ patients, regardless of HER2 status, that received chemotherapy as part of their primary breast cancer treatments, 564 (42.9%) had died by the end of the study follow-up period, of which 334 (59.2%) were recorded as caused by breast cancer (Table 1). All patients who received chemotherapy were included, regardless of chemotherapy type (neo-adjuvant or adjuvant). On inspection of the Kaplan–Meier survival curve, there was no difference in survival between ER+ IDC and ER+ ILC in the first 5 years after diagnosis, but after 5 years, there was worse survival in ILC (Figure 3a). However, there was only weak evidence (*p* = 0.088) to indicate a possible survival disadvantage in ILC compared to IDC in the univariate Cox proportional-hazards regression analysis (HR: 1.24, 95% CI: 0.97, 1.58) (Table 4).

Once adjusted for tumour size, nodal spread, grade, age, and HER2 status, there was evidence of a borderline difference in survival after chemotherapy in ER+ ILC vs. ER+ IDC (HR: 1.30, 95% CI: 1.00, 1.70) (Table 4). The model was stratified by follow-up <5 years or ≥5 years due to the difference in survival displayed in the Kaplan–Meier survival curve, and the proportional-hazards assumption was assessed by visual inspection of a log–log plot (Figure S3). Lines on the log–log plot did not cross or meet over the follow-up period, indicating proportional hazards, and therefore it was assumed that the proportional-hazards assumption was not violated.

As IDC is more likely to be HER2 positive, and therefore more likely to benefit from chemotherapy than HER2-negative disease, we repeated the analysis excluding all HER2+ and HER2-missing cases in order to form a comparable control group against ILC. This left 784 participants, of whom 298 (38.0%) had died by the end of the study follow-up, with 171 of these deaths (57.4%) listed with breast cancer as cause of death. Here, the Kaplan–Meier survival curve displayed worse survival in ILC compared to IDC for the follow-up period (Figure 3b). Similar distributions were observed in the patient demographics (Table S2) and clinicopathological characteristics (Table S3) in ER+HER2− cancers, with ILC presenting as larger tumours, with more extensive nodal spread. When separated into diagnoses before and after 1995, worse survival over the follow-up period was only observed in diagnoses after 1995, whereas before 1995 similar survival was observed between IDC and ILC for the first 10 years, after which survival was worse in ILC (Figure 3c).

The univariate Cox proportional-hazards model showed an estimated 46% worse overall survival risk in ER+HER2− ILC compared to ER+HER2− IDC cases after chemotherapy (*p* = 0.009). Evidence of this association was still present after adjusting for age at diagnosis, tumour grade, tumour size, and lymph node spread (*p* = 0.021), with a 46% higher risk of death in ILC after adjustment (95% CI: 1.06, 2.01) (Table 5). The proportional-hazards assumption was determined to be valid after inspection of a log–log plot (Figure S4) with lines not crossing or meeting over the follow-up period.

A total of 582 cases with at least 5 years survival time had their primary cancer treated with chemotherapy, and clear evidence of worse survival was still observed in ER+HER2− ILC compared to ER+HER2− IDC (HR: 1.60, 95% CI: 1.05, 2.44) from the multivariate Cox proportional-hazards regression analysis (Table S4). This analysis was also repeated for breast cancer-specific survival (BCSS) where there was still strong evidence of worse survival in ILC (HR: 1.67, 95% CI: 1.10, 2.53) (Table S5); however, this analysis censors diagnoses with a missing cause of death.

### 3.3. Recurrences in Ductal and Lobular Breast Cancer

**Figure 4:**
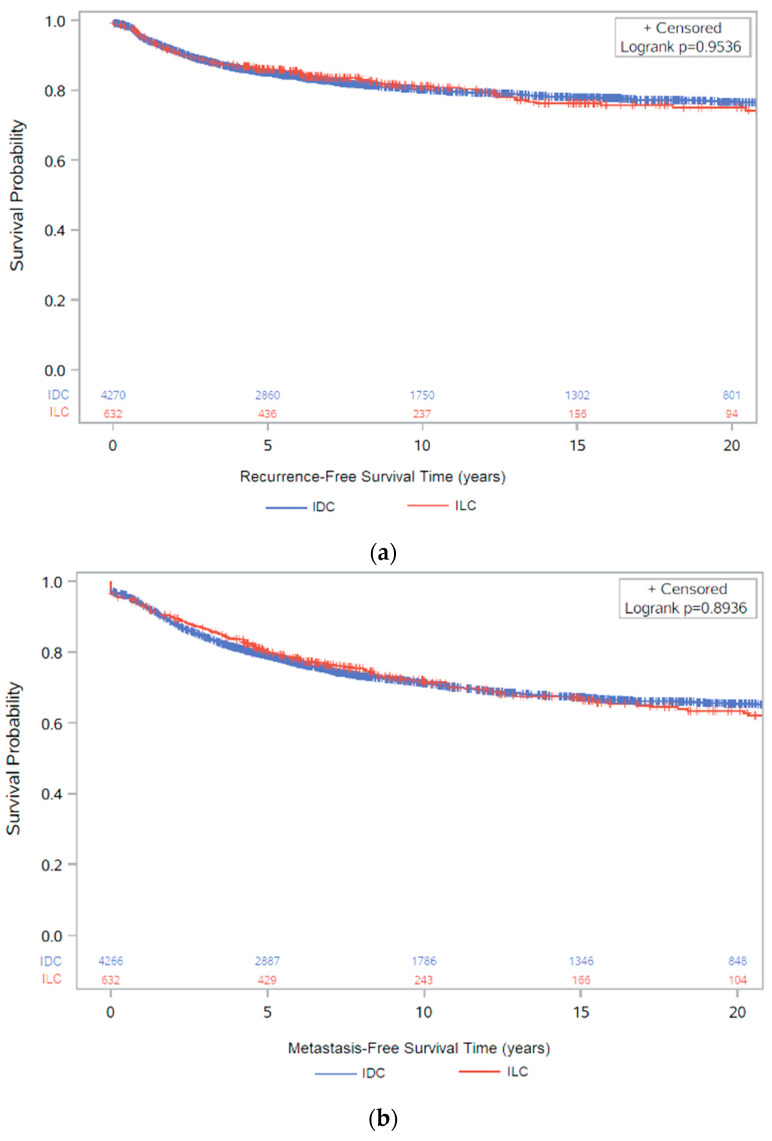
Kaplan–Meier survival analysis (with number of subjects at risk) of: (**a**) recurrence-free survival between ER+ IDC and ER+ ILC (*p* = 0.9536); (**b**) metastasis-free survival between ER+ IDC and ER+ ILC (*p* = 0.9106). These figures show results for patients with ER+ disease, irrespective of HER2 status.

A total of 811 (19.0%) ER+ IDC patients had a local recurrence of breast cancer, and 1264 (29.6%) had a distant recurrence. The recurrence rate was similar in ER+ ILC with 120 (19.0%) and 183 (28.9%) local and distant recurrences, respectively. There were no differences between ILC and IDC in recurrence-free survival (either local recurrence or distant metastasis) or metastasis-free survival (Figure 4) and the Cox proportional-hazards models gave similar findings (Table S6).

For both ER+ IDC and ER+ ILC, the majority of recurrences (local and distant) occurred within 5 years of breast cancer diagnosis, with 1104 ER+ IDC and 155 ER+ ILC within 5 years versus 415 ER+ IDC and 71 ER+ ILC after 5 years (Table S7). Clinicopathological features associated with early relapse were similar for both subtypes and included: age > 50 at diagnosis, larger tumour size, and nodal involvement, and for IDC, grade 3 tumours.

Bone was the most frequently reported site of metastases in both ER+ IDC and ER+ ILC. Liver metastases were more commonly reported in IDC patients compared to the ILC group (32.9 vs. 21.9%, *p* = 0.003), as were lung metastases (44.5 vs. 24.0%, *p* < 0.001) (Table 6). However, ILC was significantly more likely to metastasise to the peritoneum (5.7 vs. 13.1%, *p* < 0.001) and less common “other” sites such as adrenal glands and ovaries compared to IDC (23.5% in ILC vs. 14.8% in IDC) (*p* = 0.003).

## 4. Discussion

The present study confirms that clinicopathological differences persist between ILC and IDC even when the analysis is confined to ER+ disease, with ILC presenting at an older age, more likely to be T2/T3 tumours, and less frequently HER2+ than IDC. Despite the larger tumour size at presentation, ILC was no more likely to present with de novo metastatic disease than ER+ IDC in our study. We also observed, as have others, that ILC was more likely to spread to the bones, peritoneum, and gastrointestinal tract than ER+ IDC and less likely to affect the lungs and liver [12,35,36,37]. Importantly, our results show evidence of worse survival in ILC compared to ER+HER2− IDC in those receiving systemic chemotherapy.

Following the publication by Pestalozzi et al. [11], which showed better survival in ER+ ILC compared to ER+ IDC at 5 years but worse survival after 10 years, there has been concern that despite ILC having phenotypically less aggressive features than IDC, the long-term outcome of ILC may be worse. Our crude survival data is similar to that presented by Pestalozzi et al.; however, when patients pre-2002 were excluded, this early survival advantage of ILC was not seen, suggesting that this may be due to more HER2−positive patients in the IDC group who had a worse survival before the routine use of targeted anti-HER2 therapy in the adjuvant setting. When standard clinicopathological covariates were included in multivariate Cox proportional-hazards models, ER+ ILC and ER+ IDC had similar survival, indicating the importance of correcting for clinicopathological factors when undertaking these analyses. This is supported by the study by Yang et al., who demonstrated that ILC and IDC patients had similar overall survival after propensity score matching [38], but not by Adachi et al., who showed that luminal ILC had worse outcomes than luminal IDC, even when tumour size, lymph node status, and histological grade were considered [39].

There has been controversy about the benefit of chemotherapy in ILC after multiple studies have observed significantly lower pCR rates in ILC compared to IDC post NACT [17,18,40,41]. In the majority of studies, this lower pCR rate is still found even when subgroups of ILC and IDC with similar receptor status are compared (Table S8), and thus cannot be accounted for by the higher number of ER- and HER2+ cases found in IDC. However, the effect of this low pCR rate on survival is not clear. It has been suggested that pCR is not prognostic in ILC, although Riba et al. did show a survival benefit for ILC cases that achieved a pCR [18]. An interesting study comparing NACT to ACT in node-positive ILC using data from the National Cancer Data Base (2004–2013) showed that NACT was associated with a worse survival in ILC compared to adjuvant chemotherapy, even after adjusting for covariates, presumably due to a delay in definitive surgery, allowing some chemoresistant tumours to progress and also allowing for a delay in starting effective endocrine therapy [42].

The low pCR rate seen in ILC means that NACT is not routinely used to try and down stage the primary tumour or axillary nodes prior to surgery, but adjuvant chemotherapy is still given in high-risk cases to treat micro-metastatic disease. In our study, ILC patients who received chemotherapy (NACT or ACT) had a worse overall survival than ER+HER2− IDC patients, even when adjusted by histological factors that affect survival (tumour size, grade, nodal spread, age at diagnosis). Other studies of ACT in ILC also support this finding (Table S8). A large Dutch study of ER+ post-menopausal women was the first to suggest that ACT does not result in a survival benefit when added to endocrine therapy in ILC, but does in IDC [19].

However, the study was difficult to interpret as HER2 status was not known for any of the patients. Subsequently, similar findings have been reported by Marmor et al. in ER+HER2− stage I–II ILC and IDC, and by Hu et al. who found no difference in overall survival in early-stage ER+HER2− ILC after ACT vs. no chemotherapy [16,20]. In contrast, de Nonneville et al. found there was a survival advantage to ACT in high-risk ER+HER2− ILC, defined as having either macroscopic lymph node involvement, or a tumour size over 20 mm and LVI, but not in low-risk ILC [43], as did Tamirisra et al. [42]. This suggests that although the majority of ILC are not chemosensitive, there is a subset of high-risk ILC that do benefit from treatment with chemotherapy. It is not clear whether this lack of sensitivity to chemotherapy is due to the low proliferative index typical of ILC, or due to inherent molecular characteristics, such as inactivation of E-cadherin leading to increased epithelial-to-mesenchymal transition and chemoresistance or a higher frequency of PIK3CA gene mutations [44,45]. Comparison of outcomes in ER+ ILC and ER+ IDC cases with similar Ki67 or Oncotype Dx scores treated with chemotherapy would help to resolve this.

Although ER+ invasive breast carcinomas are generally considered to relapse later than ER- breast cancers, the majority of relapses in both the ER+ IDC and ILC subgroups occurred within 5 years of diagnosis. In the present study there were 598 ER+ IDC patients (14%) and 75 ER+ ILC patients (12%) who died within 5 years of diagnosis with a distant metastasis recorded. Molecular studies have shown that somatic HER2 mutations may be associated with poor survival outcomes in HER2-negative breast cancers [46]. A recent study has shown that mutations in the tyrosine kinase domain of HER2 were enriched in ILC vs. IDC cases (5.7 vs. 1.4%, *p* < 0.0001) and associated with worse survival in ILC tumours compared to ILC HER2 wild type (66  vs. 211 months, *p*  =  0.0001) but not in IDC (159 vs. 166 months, *p* = 0.733) [47]. HER2 mutations may therefore be a potential biomarker for early relapse in ILC as well as identifying those who would are likely to benefit from adjuvant neratinib.

## 5. Conclusions

Overall, this retrospective study identified distinct clinicopathological features of ER+ invasive lobular carcinoma and suggested a worsened prognosis of this histological subtype after treatment with chemotherapy, suggesting that many ILC are less sensitive to chemotherapy than IDC. Thus, other approaches need to be considered, such as the use of CDK4/6 inhibitors in the adjuvant setting which can be given with endocrine therapy. However, thus far there have been inconclusive results on the effectiveness of CDK4/6 inhibitors in early-stage breast cancers [48], although few of the studies have performed analyses to look at the ILC subgroup alone. Further investigation into survival after either adjuvant or neo-adjuvant chemotherapy are needed in a larger and stage-matched population to confirm the findings of this study. However, such results indicate that treatment guidelines for ILC should be reviewed independently of IDC, with the view of improving clinical outcomes and prognosis. It is also imperative that biomarkers that identify those ILC patients that do benefit from chemotherapy are developed, as it is not clear whether molecular tests such as Oncotype DX currently perform this effectively [49].

## Figures and Tables

**Table 1:** Demographics of patients diagnosed with ER+ IDC and ER+ ILC *.

Characteristics	ER+ IDC (%)	ER+ ILC (%)	*p*-Value (*x*^2^)	ER+ IDC (%)	ER+ ILC (%)	*p*-Value (*x*^2^)
	N = 4276	N = 633		N = 1159	N = 156	
	All ER+ patients			In those who received neo/adjuvant chemotherapy		
**Age at diagnosis ^1^**	57.2 (±13.7)	59.6 (±13.0)	<0.001	48.6 (±10.5)	51.2 (±9.8)	0.037
**Age at diagnosis**			<0.001			0.045
Below 50	1341 (31.4)	154 (24.3)		641 (55.3)	73 (46.8)	
Above 50	2935 (68.6)	479 (75.7)		518 (44.7)	83 (53.2)	
**All-cause deaths**	2467 (57.7)	364 (57.5)	0.928	489 (42.2)	75 (48.1)	0.163
Breast cancer	1179 (27.6)	176 (27.8)		289 (24.9)	45 (28.9)	
Other/missing death causes	1288 (30.1)	188 (29.7)		200 (17.2)	30 (19.2)	
**Follow-up time ^1^**	8.4 (± 9.6)	8.3 (± 8.9)	0.061	7.8 (± 8.8)	6.9 (± 8.3)	0.159
**Follow-up time**			0.043			0.420
<5 years	1114 (26.1)	156 (24.6)		309 (26.6)	43 (27.6)	
5–10 years	1235 (28.9)	216 (34.1)		374 (32.3)	59 (37.8)	
10–20 years	1043 (24.4)	149 (23.5)		274 (23.6)	30 (19.2)	
20 years +	884 (20.7)	112 (17.7)		202 (17.4)	24 (15.4)	
**Family History**			0.232			0.389
No family history	2161 (50.5)	299 (47.2)		493 (42.5)	57 (36.5)	
1st or 2nd degree, or both	632 (14.8)	98 (15.5)		157 (13.6)	23 (14.7)	
3rd degree	11 (0.3)	0 (0)		6 (0.5)	0 (0)	
Missing	1472 (34.4)	236 (37.3)		503 (43.4)	76 (48.7)	
**Ethnicity**			0.055			0.080
White	1383 (32.3)	235 (37.1)		426 (36.8)	74 (47.4)	
Black	265 (6.2)	31 (4.9)		129 (11.1)	10 (6.4)	
Asian	67 (1.6)	5 (0.8)		26 (2.2)	1 (0.6)	
Mixed	20 (0.5)	5 (0.8)		9 (0.8)	2 (1.3)	
Other	59 (1.4)	5 (0.8)		26 (2.2)	3 (1.9)	
Missing	2482 (58.0)	352 (55.6)		543 (46.9)	66 (42.3)	
**Number of births**			0.267			0.212
0	639 (14.9)	77 (12.2)		136 (11.7)	14 (9.0)	
1–2	1291 (30.2)	192 (30.3)		312 (26.9)	39 (25.0)	
3–4	576 (13.5)	88 (13.9)		141 (12.2)	20 (12.8)	
5+	107 (2.5)	22 (3.5)		21 (1.8)	7 (4.5)	
Unknown/missing	1663 (38.9)	254 (40.1)		549 (47.4)	76 (48.7)	

* This table shows results for patients with ER+ disease, irrespective of HER2 status; ^1^ All continuous variables are displayed as median values (±standard deviation).

**Table 2:** Histological tumour characteristics of patients diagnosed with ER+ IDC and ER+ ILC *.

Characteristics	ER+ IDC (%)	ER+ ILC (%)	*p*-Value (*x*^2^)	ER+ IDC (%)	ER+ ILC (%)	*p*-Value (*x*^2^)
	N = 4276	N = 633		N = 1159	N = 156	
	All ER+ patients			In those who received primary chemotherapy		
**Chemotherapy**			0.056			0.454
Neo-adjuvant (NACT)	325 (7.6)	62 (9.8)		195 (16.8)	30 (19.2)	
Adjuvant (ACT)	969 (22.7)	124 (19.6)		964 (83.2)	126 (80.8)	
**HER2 Status ^3^**			<0.001			<0.001
Negative	2267 (53.0)	420 (66.4)		656 (56.6)	128 (82.1)	
Positive	521 (12.2)	35 (5.5)		239 (20.6)	17 (10.9)	
Missing	1488 (34.8)	178 (28.1)		264 (22.8)	11 (7.0)	
**Tumour size (mm) ^1^**	20.0 (±17.7)	22.0 (±24.0)	<0.001	22 (±18.6)	30 (±27.8)	<0.001
**T Stage**			<0.001			<0.001
T1 (1–20 mm)	2179 (51.0)	269 (42.5)		478 (41.2)	38 (24.4)	
T2 (20–50 mm)	1416 (33.1)	243 (38.4)		473 (40.8)	72 (46.2)	
T3 (50 mm+)	173 (4.0)	64 (10.1)		85 (7.3)	25 (16.0)	
T4	341 (8.0)	44 (6.9)		99 (8.5)	19 (12.2)	
Missing	167 (3.9)	13 (2.1)		24 (2.1)	2 (1.3)	
**N Stage**			0.001			0.006
N0	1730 (40.5)	276 (43.6)		247 (21.3)	30 (19.2)	
N1	1182 (27.6)	142 (22.4)		512 (44.2)	54 (34.6)	
N2	372 (8.7)	51 (8.1)		172 (14.8)	25 (16.0)	
N3	220 (5.1)	54 (8.5)		115 (9.9)	30 (19.2)	
Missing	772 (18.1)	110 (17.4)		113 (9.8)	17 (10.9)	
**Grade ^2^**			<0.001			<0.001
Grade 1	566 (13.2)	14 (2.2)		71 (6.1)	3 (1.9)	
Grade 2	2020 (47.2)	563 (88.9)		459 (39.6)	131 (84.0)	
Grade 3	1449 (33.9)	56 (8.9)		571 (49.3)	22 (14.1)	
Missing	241 (5.6)	0 (0)		58 (5.0)	0 (0)	
**Metastatic on diagnosis**	190 (4.4)	31 (4.9)	0.607	51 (4.4)	7 (4.5)	0.960

* This table shows results for patients with ER+ disease, irrespective of HER2 status. ^1^ All continuous variables are displayed as median values (±standard deviation); ^2^ ILC with missing grade data were assumed to be grade 2; ^3^ HER2 status was missing for many cases due to lack of routine testing before 2006.

**Table 3:** Cox proportional-hazards models for overall survival following ER+ IDC or ER+ ILC (N = 4909) *.

Variables Included		OS HR (95% CI)	*p*-Value
Crude			
**Invasive type**	IDC	1 ^reference^	
	ILC	1.05 (0.94, 1.18)	0.356
Multivariate ^1^			
**Invasive type**	IDC	1 ^reference^	
	ILC	0.94 (0.83, 1.04)	0.269
**Age at diagnosis**	<50	1 ^reference^	
	>50	1.31 (1.19, 1.44)	<0.001
**Grade**	Grade 1	1 ^reference^	
	Grade 2	1.14 (1.00, 1.30)	0.046
	Grade 3	1.33 (1.16, 1.52)	<0.001
	Missing	0.63 (0.50, 0.81)	<0.001
**Tumour size (TNM)**	T1 (1–20 mm)	1 ^reference^	
	T2 (20–50 mm)	1.14 (1.04, 1.24)	0.003
	T3 (50 mm+)	1.13 (0.95, 1.36)	0.165
	T4	1.36 (1.18, 1.55)	<0.001
	Missing	1.00 (0.81, 1.24)	0.975
**Nodal spread (TNM)**	N0	1 ^reference^	
	N1	1.37 (1.24, 1.52)	<0.001
	N2	1.66 (1.45, 1.92)	<0.001
	N3	2.24 (1.91, 2.62)	<0.001
	Missing	1.86 (1.66, 2.08)	<0.001
**HER2 Status**	Negative	1 ^reference^	
	Positive	1.28 (1.13, 1.45)	<0.001
	Missing	1.50 (1.39, 1.63)	<0.001
**Chemotherapy**	No chemotherapy	1 ^reference^	
	Neo-adjuvant	0.40 (0.33, 0.49)	<0.001
	Adjuvant	0.69 (0.62, 0.77)	<0.001

* This table shows results for patients with ER+ disease, irrespective of HER2 status. ^1^ Stratified by follow-up time.

**Table 4:** Cox proportional-hazards models for overall survival after ER+ IDC or ER+ ILC following chemotherapy (N = 1315) *.

Variables Included		OS HR (95% CI)	*p*-Value
Crude			
**Invasive Type**	IDC	1 ^reference^	
	ILC	1.24 (0.97, 1.58)	0.088
Multivariate ^1^			
**Invasive type**	IDC	1 ^reference.^	
	ILC	1.30 (1.00, 1.70)	0.050
**Age**	<50	1 ^reference.^	
	>50	1.38 (1.16, 1.63)	<0.001
**Grade**	Grade 1	1 ^reference.^	
	Grade 2	1.11 (0.74, 1.66)	0.618
	Grade 3	1.54 (1.03, 2.32)	0.037
	Missing	0.95 (0.53, 1.69)	0.855
**Tumour size (TNM)**	T1 (1–20 mm)	1 ^reference.^	
	T2 (20–50 mm)	1.21 (0.98, 1.48)	0.070
	T3 (50 mm+)	1.31 (0.94, 1.81)	0.106
	T4	1.29 (0.97, 1.72)	0.085
	Missing	1.40 (0.78, 2.50)	0.261
**Nodal spread (TNM)**	N0	1 ^reference.^	
	N1	2.30 (1.63, 3.25)	<0.001
	N2	3.29 (2.26, 4.81)	<0.001
	N3	5.27 (3.62, 7.66)	<0.001
	Missing	5.73 (3.83, 8.57)	<0.001
**HER2 Status**	Negative	1 ^reference.^	
	Positive	1.17 (0.93, 1.47)	0.184
	Missing	1.52 (1.24, 1.87)	<0.001

* This table shows results for patients with ER+ disease, irrespective of HER2 status. ^1^ Stratified by follow-up time (<5 years or ≥5 years).

**Table 5:** Cox proportional-hazards models for overall survival after ER+HER2− IDC or ILC following chemotherapy (N = 784) *.

Variables Included		OS HR (95% CI)	*p*-Value
Crude			
**Invasive type**	IDC	1 ^reference^	
	ILC	1.46 (1.10, 1.93)	0.009
Multivariate			
**Invasive type**	IDC	1 ^reference.^	
	ILC	1.46 (1.06, 2.01)	0.021
**Age**	<50	1 ^reference.^	
	>50	1.41 (1.12, 1.77)	0.004
**Grade**	Grade 1	1 ^reference.^	
	Grade 2	2.15 (1.12, 4.13)	0.022
	Grade 3	3.35 (1.75, 6.39)	<0.001
	Missing	3.37 (1.18, 9.57)	0.023
**Tumour size (TNM)**	T1 (1–20 mm)	1 ^reference.^	
	T2 (20–50 mm)	1.39 (1.05, 1.83)	0.023
	T3 (50 mm+)	1.50 (0.98, 2.29)	0.061
	T4	2.10 (1.40, 3.16)	<0.001
	Missing	2.86 (0.96, 8.55)	0.060
**Nodal spread (TNM)**	N0	1 ^reference.^	
	N1	2.07 (1.32, 3.24)	0.001
	N2	3.25 (2.02, 5.25)	<0.001
	N3	6.41 (3.99, 10.31)	<0.001
	Missing	4.76 (2.76, 8.23)	<0.001

* This table shows results for patients with ER+ and HER2− disease.

**Table 6:** Characteristics of distant metastatic recurrences in ER+ IDC and ER+ ILC (N = 1447) *.

Metastatic Characteristics	IDC (%)	ILC (%)	*p*-Value (*x*^2^)
	N = 1264	N = 183	
**Age at metastasis** ^1^	60.9 (±13.3)	61.0 (±12.5)	0.154
**Time to death after metastasis** ^1^	1.8 (±3.6)	1.9 (±4.5)	0.385
**Recurrence-free survival time** ^1^	3.6 (±5.8)	4.8 (±6.0)	0.206
**Metastasis-free survival time** ^1^	2.8 (±4.8)	3.4 (±4.6)	0.462
**Site of distant metastasis**			
Bone	856 (67.7)	135 (73.8)	0.100
Lung	562 (44.5)	44 (24.0)	<0.001
Liver	416 (32.9)	40 (21.9)	0.003
Lymph nodes	262 (20.7)	32 (17.5)	0.308
Cutaneous	177 (14.0)	34 (18.6)	0.101
Brain	157 (12.4)	12 (6.7)	0.021
Peritoneal/GI	72 (5.7)	24 (13.1)	<0.001
Other (adrenal glands, ovary)	187 (14.8)	43 (23.5)	0.003

* This table shows results for patients with ER+ disease, irrespective of HER2 status; ^1^ All continuous variables are displayed as median values (±standard deviation).

## Data Availability

Data used in this study are not publicly available for ethical reasons, due to the use of patient data.

## Supplementary Materials

The following are available online at https://www.mdpi.com/article/10.3390/cancers13123036/s1: Figure S1: Diagnosis of ductal (ER+ IDC) and lobular (ER+ ILC) breast cancer between 1975–2016, irrespective of HER2 status, Figure S2: Log-log plot for survival in ER+ breast cancer by follow-up time, adjusted by breast cancer type (IDC or ILC) (N = 4909), irrespective of HER2 status, Figure S3: Log-log plot of survival in ER+ IDC and ER+ ILC stratified by 5 years follow-up, adjusted by breast cancer type (IDC or ILC) (N = 1315), irrespective of HER2 status, Figure S4: Log–log plot of survival in ER+HER2− IDC and ER+HER2− ILC (N = 784), Table S1: Landmark analysis: Cox proportional-hazards models for overall survival after ER+ IDC or ILC, in those with at least 5 years follow-up (N = 3639), Table S2: Demographics of patients diagnosed with ER+ IDC and ER+ ILC, who received chemotherapy treatment as part of their primary treatment, before and after HER2 exclusions, Table S3: Histological tumour characteristics of patients diagnosed with ER+ IDC and ER+ ILC, who received chemotherapy treatment as part of their primary treatment, before and after HER2 exclusions, Table S4: Landmark analysis—Cox proportional-hazards models for overall survival after ER+HER2− IDC or ILC following chemotherapy, in those with at least 5 years follow-up (N = 582), Table S5. Cox proportional-hazards models for breast cancer-specific survival after ER+HER2− IDC or ILC following chemotherapy, in those with at least 5 years follow-up (N = 784), Table S6: Recurrence-free survival (RFS) and metastasis-free survival (MFS) in ER+ IDC and ER+ ILC (N = 4909), Table S7: Histological tumour characteristics of patients diagnosed with ER+ IDC and ER+ ILC who had a local or distant recurrence within 5 years of diagnosis (N = 1259) compared to those who had a local or distant recurrence at least 5 years after diagnosis (N = 486), Table S8: Summary of results from studies investigating outcomes after chemotherapy in IDC and ILC.
