# Supplementary material for: Survival Outcomes in Invasive Lobular Carcinoma Compared to Oestrogen Receptor-Positive Invasive Ductal Carcinoma
Source: Cancers (Basel). 2021 Jun 18;13(12):3036. doi: 10.3390/cancers13123036 (PMC8234044; doi:10.3390/cancers13123036)
Supplement: Supplementary file 1 [file cancers-13-03036-s001.zip › cancers-1224578-supplementary.pdf]

Table S1. Landmark analysis: Cox proportional-hazards models for overall survival after ER+ IDC or ILC, in those with at least 5 years follow-up (N=3639)*.

Variables included		HR (95% CI)	p-value
	Crude		
**Invasive type**	IDC	1 reference	
	ILC	1.15 (1.00, 1.32)	0.042
	Multivariate[1]		
**Invasive type**	IDC	1 reference	
	ILC	0.97 (0.83, 1.12)	0.655
**Age**	<50	1 reference	
	>50	1.28 (1.14, 1.43)	<0.001
**Grade**	Grade 1	1 reference	
	Grade 2	1.17 (1.01, 1.35)	0.037
	Grade 3	1.28 (1.09, 1.50)	0.003
	Grade missing	0.57 (0.41, 0.79)	0.001
**Tumour size (TNM)**	T1 *(1-20mm)*	1 reference	
	T2 *(20-50mm)*	1.01 (0.91, 1.13)	0.827
	T3 *(50mm+)*	0.86 (0.66, 1.11)	0.236
	T4	1.40 (1.15, 1.71)	0.001
	Missing	0.87 (0.65, 1.16)	0.349
**Nodal spread (TNM)**	N0	1 reference	
	N1	1.49 (1.32, 1.67)	<0.001
	N2	1.71 (1.44, 2.03)	<0.001
	N3	2.36 (1.89, 2.95)	<0.001
	Missing	1.69 (1.46, 1.95)	<0.001
**Chemotherapy**	None	1 reference	
	NACT	0.44 (0.33, 0.59)	<0.001
	ACT	0.52 (0.45, 0.60)	<0.001
**HER2 status**	Negative	1 reference	
	Positive	1.23 (1.04, 1.45)	0.016
	Missing	1.48 (1.33, 1.63)	<0.001

* This table shows results for patients with ER+ disease, irrespective of HER2 status.
[1]Stratified by follow-up time.

Table S2. Demographics of patients diagnosed with ER+ IDC and ER+ ILC, who received chemotherapy treatment as part of their primary treatment, before* and after◊ HER2 exclusions.

Characteristics	ER+ IDC* (%)	ER+ ILC* (%)	p-value (x^2)	ER+ HER2- IDC◊ (%)	ER+ HER2- ILC◊ (%)	p-value (x^2)
	N=1159	N=156		N=656	N=128	
**Age at diagnosis** [1]	48.6 (±10.5)	51.2 (±9.8)	0.037	48.5 (±9.9)	50.8 (±9.1)	0.017
**Age at diagnosis**			0.045			0.085
Below 50	641 (55.3)	73 (46.8)		367 (55.9)	61 (47.7)	
Above 50	518 (44.7)	83 (53.2)		289 (44.1)	67 (52.3)	
**All-cause deaths**	489 (42.2)	75 (48.1)	0.163	237 (36.1)	61 (47.7)	0.014
Breast cancer	289 (24.9)	45 (28.9)		134 (20.4)	37 (28.9)	
Other/missing death causes	200 (17.2)	30 (19.2)		103 (15.7)	24 (18.8)	
**Follow-up time** [1]	7.8 (±8.8)	6.9 (±8.3)	0.159	8.1 (±7.9)	7.0 (±8.0)	0.159
**Follow-up time**			0.420			0.376
< 5 years	309 (26.6)	43 (27.6)		167 (25.5)	35 (27.3)	
5 – 10 years	374 (32.3)	59 (37.8)		217 (33.1)	50 (39.1)	
10 – 20 years	274 (23.6)	30 (19.2)		175 (26.7)	26 (20.3)	
20 years +	202 (17.4)	24 (15.4)		97 (15.8)	17 (13.3)	
**Family History**			0.389			0.772
No family history	493 (42.5)	57 (36.5)		232 (35.4)	45 (35.2)	
1st or 2nd degree, or both	157 (13.6)	23 (14.7)		97 (14.8)	16 (12.5)	
3rd degree	6 (0.5)	0 (0)		6 (0.9)	0 (0)	
Missing	503 (43.4)	76 (48.7)		321 (48.9)	67 (52.3)	
**Ethnicity**			0.080			0.260
White	426 (36.8)	74 (47.4)		272 (41.5)	61 (47.7)	
Black	129 (11.1)	10 (6.4)		75 (11.4)	8 (6.3)	
Asian	26 (2.2)	1 (0.6)		15 (2.3)	1 (0.8)	
Mixed	9 (0.8)	2 (1.3)		4 (0.6)	2 (1.6)	
Other	26 (2.2)	3 (1.9)		16 (2.4)	3 (2.3)	
Missing	543 (46.9)	66 (42.3)		274 (41.8)	53 (41.4)	
**Number of births**			0.212			0.397
0	136 (11.7)	14 (9.0)		68 (10.4)	13 (10.2)	
1 - 2	312 (26.9)	39 (25.0)		161 (24.5)	29 (22.7)	
3 - 4	141 (12.2)	20 (12.8)		68 (10.4)	14 (10.9)	
5+	21 (1.8)	7 (4.5)		9 (1.4)	5 (3.9)	
Unknown/missing	549 (47.4)	76 (48.7)		350 (53.3)	67 (52.3)	

* These are results for patients with ER+ disease, irrespective of HER2 status. ◊ This are results for patients with ER+ and HER2- disease. [1] All continuous variables are displayed as median values (± standard deviation).

Table S3. Histological tumour characteristics of patients diagnosed with ER+ IDC and ER+ ILC, who received chemotherapy treatment as part of their primary treatment, before* and after◊ HER2 exclusions.

**Characteristics**	**ER+ IDC* (%)**	**ER+ ILC* (%)**	**p-value (x^2)**	**ER+HER2- IDC◊ (%)**	**ER+HER2- ILC◊ (%)**	**p-value (x^2)**
	N=1159	N=156		N=656	N=128	
**Chemotherapy**			0.454			1.000
NACT	195 (16.8)	30 (19.2)		123 (18.7)	24 (18.7)	
ACT	964 (83.2)	126 (80.8)		533 (81.3)	104 (81.3)	
**HER2 Status**[1]			<0.001			
Negative	656 (56.6)	128 (82.1)		656 (100.0)	128 (100)	
Positive	239 (20.6)	17 (10.9)		-	-	
Missing	264 (22.8)	11 (7.0)		0 (0)	0 (0)	
**Tumour size (mm)**[2]	22 (±18.6)	30 (±27.8)	<0.001	22.5 (±18.8)	30.5 (±28.9)	<0.001
**T Stage**			<0.001			<0.001
T1 *(1-20mm)*	478 (41.2)	38 (24.4)		264 (40.2)	27 (21.1)	
T2 *(20-50mm)*	473 (40.8)	72 (46.2)		292 (44.5)	62 (48.4)	
T3 *(50mm+)*	85 (7.3)	25 (16.0)		47 (7.2)	24 (18.8)	
T4	99 (8.5)	19 (12.2)		42 (6.4)	14 (10.9)	
Missing	24 (2.1)	2 (1.3)		11 (1.7)	1 (0.8)	
**N Stage**			0.006			0.004
N0	247 (21.3)	30 (19.2)		143 (21.8)	25 (19.5)	
N1	512 (44.2)	54 (34.6)		303 (46.2)	43 (33.6)	
N2	172 (14.8)	25 (16.0)		97 (14.8)	21 (16.4)	
N3	115 (9.9)	30 (19.2)		66 (10.1)	26 (20.3)	
Missing	113 (9.8)	17 (10.9)		47 (7.2)	13 (10.2)	
**Grade** [3]			<0.001			<0.001
Grade 1	71 (6.1)	3 (1.9)		44 (6.7)	3 (2.3)	
Grade 2	459 (39.6)	131 (84.0)		249 (38.0)	107 (83.6)	
Grade 3	571 (49.3)	22 (14.1)		335 (51.1)	18 (14.1)	
Missing	58 (5.0)	0 (0)		28 (4.3)	0 (0)	
**Metastatic on diagnosis**	51 (4.4)	7 (4.5)	0.960	17 (2.6)	7 (5.5)	0.084

* These are results for patients with ER+ disease, irrespective of HER2 status. ◊ This are results for patients with ER+ and HER2- disease. [1] HER2 positive patients and HER2 missing patients were excluded from chemotherapy analysis. [2] All continuous variables are displayed as median values (± standard deviation). [3] ILC with missing grade data were assumed to be Grade 2.

Table S4. Landmark analysis – Cox proportional-hazards models for overall survival after ER+HER2- IDC or ILC following chemotherapy, in those with at least 5 years follow-up (N=582)*.

**Variables included**		**HR (95% CI)**	**p-value**
		Crude	
**Invasive type**	IDC	1 reference	
	ILC	1.65 (1.14, 2.39)	0.008
		Multivariate	
**Invasive type**	IDC	1 reference	
	ILC	1.60 (1.05, 2.44)	0.029
**Age**	<50	1 reference	
	>50	1.24 (0.91, 1.69)	0.183
**Grade**	Grade 1	1 reference	
	Grade 2	2.48 (1.13, 5.46)	0.024
	Grade 3	3.09 (1.41, 6.76)	0.005
	Grade missing	11.78 (3.31, 41.95)	<0.001
**Tumour size (TNM)**	T1 *(1-20mm)*	1 reference	
	T2 *(20-50mm)*	1.23 (0.86, 1.77)	0.255
	T3 *(50mm+)*	1.50 (0.86, 2.62)	0.150
	T4	2.17 (1.23, 3.83)	0.008
	Missing	omitted	omitted
**Nodal spread (TNM)**	N0	1 reference	
	N1	2.00 (1.12, 3.58)	0.020
	N2	3.15 (1.69, 5.88)	<0.001
	N3	6.78 (3.59, 12.80)	<0.001
	Missing	2.31 (0.97, 5.14)	0.059

* This table shows results for patients with ER+ and HER- disease.

Table S5. Cox proportional-hazards models for breast cancer specific survival after ER+HER2- IDC or ILC following chemotherapy, in those with at least 5 years follow-up (N=784)*.

**Variables included**		**HR (95% CI)**	**p-value**
	Crude		
**Invasive type**	IDC	1 reference	
	ILC	1.54 (1.07, 2.21)	0.021
	Multivariate		
**Invasive type**	IDC	1 reference	
	ILC	1.67 (1.10, 2.53)	0.017
**Age**	<50	1 reference	
	>50	1.27 (0.94, 1.72)	0.123
**Grade**	Grade 1	1 reference	
	Grade 2	2.00 (0.80, 5.01)	0.141
	Grade 3	3.75 (1.52, 9.28)	0.004
	Grade missing	2.20 (0.42, 11.53)	0.350
**Tumour size (TNM)**	T1 *(1-20mm)*	1 reference	
	T2 *(20-50mm)*	1.39 (0.96, 2.01)	0.084
	T3 *(50mm+)*	1.14 (0.64, 2.02)	0.659
	T4	1.83 (1.09, 3.09)	0.023
	Missing	omitted	omitted
**Nodal spread (TNM)**	N0	1 reference	
	N1	3.00 (1.48, 6.10)	0.002
	N2	5.92 (2.85, 12.29)	<0.001
	N3	10.28 (4.95, 21.34)	<0.001
	Missing	10.64 (4.84, 23.35)	<0.001

* This table shows results for patients with ER+ and HER- disease.

Table S6. Recurrence-free survival (RFS) and metastasis-free survival (MFS) in ER+ IDC and ER+ ILC (N=4909)*.

**Variables included**		**RFS HR (95% CI)**	**p-value**	**MFS HR (95% CI)**	**p-value**
		Crude			
**Invasive type**	IDC	1 reference		1 reference	
	ILC	0.99 (0.81, 1.21)	0.939	0.96 (0.81, 1.13)	0.616
		Multivariate			
**Invasive type**	IDC	1 reference		1 reference	
	ILC	0.91 (0.74, 1.11)	0.341	0.90 (0.76, 1.07)	0.243
**Age**[2]	<50	1 reference		1 reference	
	>50	0.68 (0.59, 0.79)	<0.001	0.94 (0.92, 0.95)	<0.001
**Grade**[1]	Grade 1	1 reference		1 reference	
	Grade 2	1.09 (1.04, 1.14)	<0.001	2.18 (1.67, 2.85)	<0.001
	Grade 3	1.09 (1.04, 1.15)	0.001	2.33 (1.78, 3.06)	<0.001
	Missing	0.73 (0.60, 0.89)	0.002	1.20 (0.83, 1.74)	0.327
**Tumour size (TNM)**[2]	T1 *(1-20mm)*	1 reference		1 reference	
	T2 *(20-50mm)*	1.24 (1.06, 1.46)	0.008	1.02 (1.00, 1.04)	0.040
	T3 *(50mm+)*	1.28 (0.95, 1.72)	0.099	1.00 (0.96, 1.05)	0.947
	T4	1.76 (1.43, 2.18)	<0.001	1.05 (1.02, 1.08)	0.002
	Missing	0.84 (0.58, 1.23)	0.370	1.08 (1.05, 1.11)	<0.001
**Nodal spread (TNM)**[1]	N0	1 reference		1 reference	
	N1	2.37 (1.93, 2.91)	<0.001	2.18 (1.86, 2.56)	<0.001
	N2	3.82 (2.98, 4.89)	<0.001	3.07 (2.51, 3.74)	<0.001
	N3	4.83 (3.69, 6.32)	<0.001	4.63 (3.77, 5.69)	<0.001
	Missing	4.50 (3.63, 5.58)	<0.001	2.53 (2.13, 3.00)	<0.001
**HER2 Status**	Negative	1 reference		1 reference	
	Positive	1.26 (1.02, 1.57)	0.033	1.61 (1.35, 1.90)	<0.001
	Missing	1.69 (1.46, 1.95)	<0.001	1.86 (1.65, 2.10)	<0.001
**Chemotherapy** [1,2]	None	1 reference		1 reference	
	NACT	0.84 (0.76, 0.92)	<0.001	0.84 (0.79, 0.89)	<0.001
	ACT	0.89 (0.86, 0.93)	<0.001	0.91 (0.89, 0.94)	<0.001

* This table shows results for patients with ER+ disease, irrespective of HER2 status. [1]Variables modelled as time-varying covariates in RFS model. [2]Variables modelled as time-varying covariates in MFS model.

Table S7. Histological tumour characteristics of patients diagnosed with ER+ IDC and ER+ ILC who had a local or distant recurrence within 5 years of diagnosis (N=1259) compared to those who had a local or distant recurrence at least 5 years after diagnosis (N = 486) *.

	ER+ IDC			ER+ ILC		
**Characteristics**	**Recurred within 5 years (%)** N=1104	**Recurred after 5 years (%)** N=415	**p-value (x^2)**	**Recurred within 5 years (%)** N=155	**Recurred after 5 years (%)** N=71	**p-value (x^2)**
**Age at diagnosis** [1]	57.0 (±14.0)	53.7 (±11.9)	<0.001	58.7 (±13.3)	52.2 (±11.3)	0.011
**Age at diagnosis**						0.301
Below 50	356 (32.3)	158 (38.1)	0.032	44 (28.4)	25 (35.2)	
Above 50	748 (67.7)	257 (61.9)		111 (71.6)	46 (64.8)	
**All-cause deaths**	975 (88.3)	356 (85.8)	0.182	140 (90.3)	64 (90.1)	0.966
Breast cancer	841 (76.2)	241 (58.1)		119 (76.8)	43 (60.6)	
Other/missing death causes	134 (12.1)	115 (27.7)		21 (13.6)	21 (29.6)	
**Follow-up time** [1]	4.4 (±5.5)	13.1 (±7.7)	<0.001	4.9 (±5.9)	13.3 (±8.0)	<0.001
**Time period of diagnosis**			<0.001			0.017
<1980	191 (17.3)	68 (16.4)		17 (11.0)	3 (4.2)	
1980-1990	431 (39.0)	171 (41.2)		70 (45.2)	39 (54.9)	
1990-2000	230 (20.8)	113 (27.2)		31 (20.0)	21 (29.6)	
2000-2010	107 (9.7)	55 (13.3)		20 (12.9)	7 (9.9)	
2010+	145 (13.1)	8 (1.9)		17 (11.0)	1 (1.4)	
**Chemotherapy for primary cancer**			<0.001			0.067
None	743 (67.3)	317 (76.4)		103 (66.4)	57 (80.3)	
NACT	94 (8.5)	16 (3.9)		15 (9.7)	2 (2.8)	
ACT	267 (24.2)	82 (19.8)		37 (23.9)	12 (16.9)	
**HER2 Status**[2]			0.094			0.072
Negative	461 (41.8)	193 (46.5)		88 (56.8)	39 (54.9)	
Positive	170 (15.4)	48 (11.6)		9 (5.8)	0 (0)	
Missing	473 (42.8)	174 (41.9)		58 (37.4)	32 (45.1)	
**Tumour size (mm)**[1]	25.0 (±23.2)	20.0 (±13.9)	<0.001	30.0 (±31.1)	24.7 (±20.8)	0.012
**T Stage**						0.141
T1 *(1-20mm)*	350 (31.7)	207 (49.9)	<0.001	34 (21.9)	24 (33.8)	
T2 *(20-50mm)*	420 (38.0)	134 (32.3)		63 (40.7)	32 (45.1)	
T3 *(50mm+)*	82 (7.4)	17 (4.1)		24 (15.5)	7 (9.9)	
T4	192 (17.4)	29 (7.0)		27 (17.4)	6 (8.5)	
Missing	60 (5.4)	28 (6.8)		7 (4.5)	2 (2.8)	
**N Stage**			<0.001			0.001
N0	174 (15.8)	127 (30.6)		21 (13.6)	24 (33.8)	
N1	289 (26.2)	135 (32.5)		29 (18.7)	17 (23.9)	
N2	132 (12.0)	50 (12.0)		18 (11.6)	10 (14.1)	
N3	124 (11.2)	26 (6.3)		33 (21.3)	6 (8.5)	
Missing	385 (34.9)	77 (18.6)		54 (34.8)	14 (19.7)	
**Grade** [3]			<0.001			0.310
Grade 1	46 (4.2)	37 (8.9)		2 (1.3)	2 (2.8)	
Grade 2	512 (46.4)	229 (55.2)		136 (87.7)	65 (91.6)	
Grade 3	501 (45.4)	129 (31.1)		17 (11.0)	4 (5.6)	
Missing	45 (4.1)	20 (4.8)		0 (0)	0 (0)	
**Metastatic on diagnosis**	141 (12.8)	5 (1.2)	<0.001	27 (17.4)	0 (0)	<0.001
**Local recurrence**	619 (56.1)	192 (46.3)	<0.001	86 (55.5)	34 (47.9)	0.288
**Distant recurrence**	926 (83.9)	338 (81.5)	0.259	129 (83.2)	54 (76.1)	0.202

* This table shows results for patients with ER+ disease, irrespective of HER2 status. [1] All continuous variables are displayed as median values (± standard deviation). [2] HER2 positive patients and HER2 missing patients were excluded from chemotherapy analysis.

Table S8. Summary of results from studies investigating outcomes after chemotherapy in IDC and ILC.

Authors	Number of cases	Comparator	Outcome	Results	pCR Rate Overall	pCR rate (ER+HER2-)	pCR rate (TNBC)	pCR rate (HR+/HER2+)	pCR rate (HR-/HER2+)
Loibl et al. 2014 [1]	9197	ILC vs non-ILC	pCR	OR: 1.81, 95%CI: 1.31-2.52	6.2% (ILC) vs 17.4% (IDC) (p=0.001)	4.5%<br>9.6%	17.5%<br>32.2%	9.1%<br>18.8%	21.7%<br>31.6%
Riba et al. 2019 [2]	384,887	pCR vs no pCR	Overall survival	(ILC) HR: 0.46, 95%CI: 0.25-0.84<br>(IDC) HR: 0.33, 95%CI: 0.30-0.37	8.7% (ILC) vs 23.2% (IDC) (p=<0.0001)	5.4%<br>11.7%	20.5%<br>27.6%	20.4%<br>26.8%	37.9%<br>38.1%
Tamirisa et al. 2018 [3]	15573	(ILC only) NACT vs ACT	Overall survival	(ILC) HR: 1.38, 95%CI: 1.25–1.52	3.4% (ILC)				
Cristofanilli et al. 2005 [4]	1034	ILC vs IDC	pCR	N/A	3% (ILC) vs 15% (IDC) (P <0 .001)	4% (Her2 unknown)<br>9%	0%<br>20%		
Delpech et al. 2013 [5]	1895	ILC vs IDC	pCR	N/A	3.5% (ILC) vs 14% (IDC) (P<0.001)				
Lips et al. 2012 [6]	676	ILC vs IDC	pCR	N/A	11% (ILC) vs. 24 % (IDC) p = 0.008	7%<br>5%	0%<br>36%	30%<br>32%	33%<br>57%
Marmor et al. 2017 [7]	36244	Endocrine vs Endocrine + ACT	10-year overall survival	(IDC) HR: 0.82 95%CI: 0.73-0.92<br>(ILC) HR: 1.18, 95%CI: 0.90-1.54					
Hu et al. 2020 [8]	12334	(ILC only) ACT vs no chemotherapy	Overall survival	HR: 0.82, 95%CI: 0.62-1.09					
Truin et al. 2012 [9]	23294	Endocrine vs Endocrine + ACT	Overall survival	(IDC) HR: 0.70 95%CI: 0.64–0.76<br>(ILC) HR: 1.00 95%CI: 0.82–1.21<br>(IDC vs ILC) HR: 1.11 95%CI: 0.94–1.30					
de Nonneville 2019 [10]	2318	(ILC only) Endocrine vs Endocrine + ACT	Overall survival	HR: 0.52, 95% CI: 0.31-0.87					

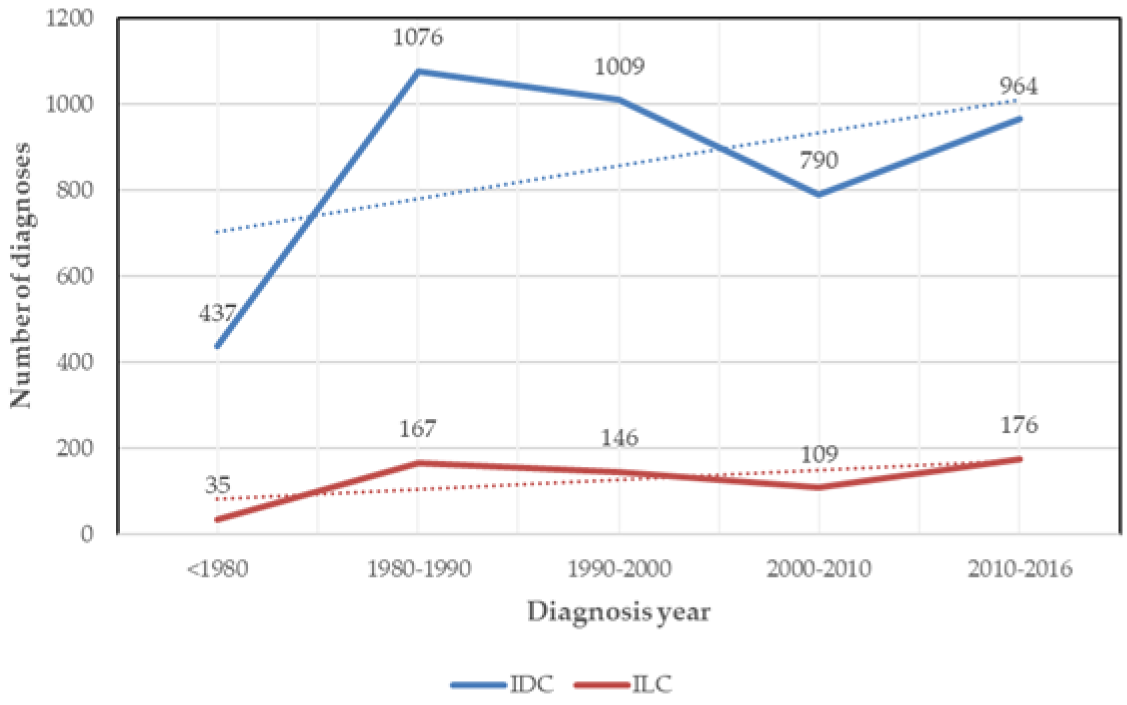

Figure S1. Diagnosis of lobular breast cancer (ILC) between 1975-2016.

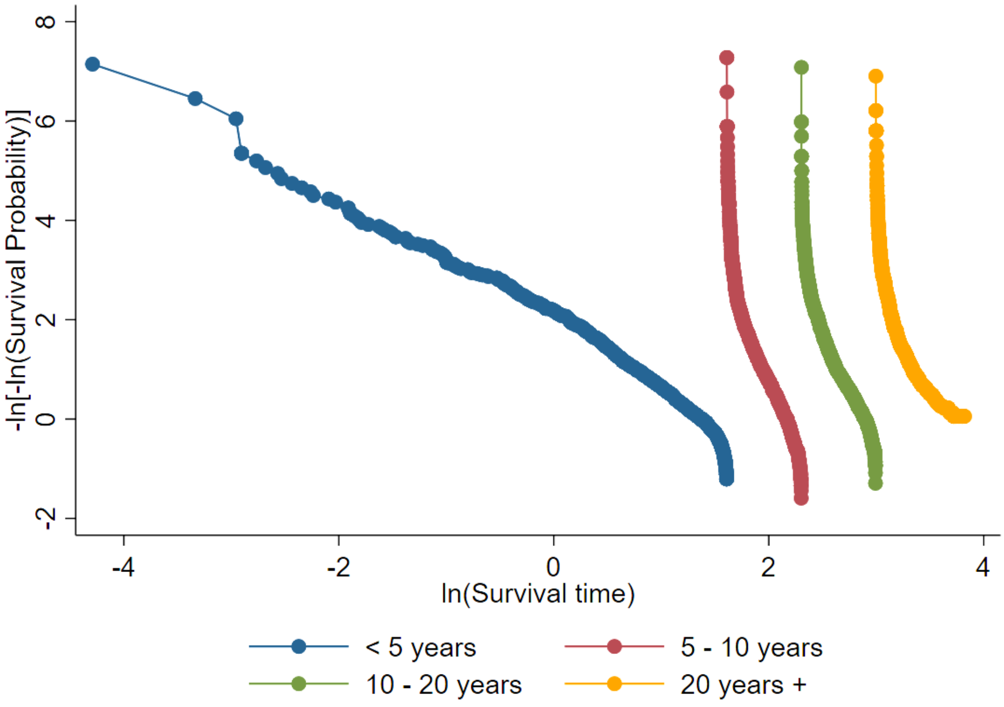

Figure S2. Log-log plot for survival in ER+ breast cancer by follow-up time, adjusted by breast cancer type (IDC or ILC) (N = 4909).

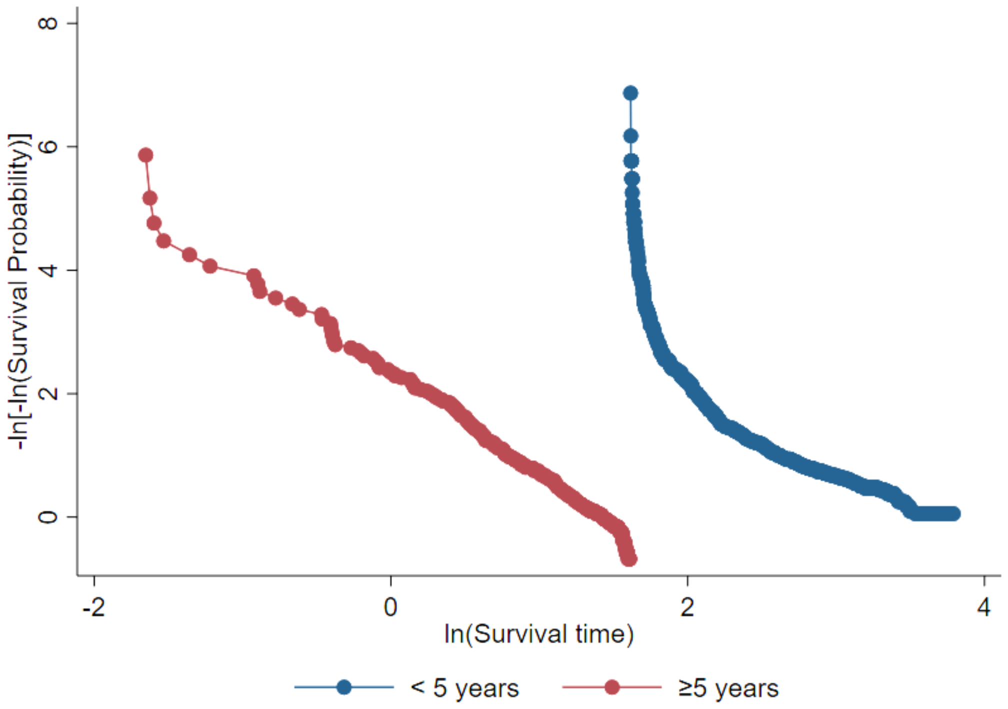

Figure S3. Log-log plot of survival in ER+ IDC and ER+ ILC stratified by 5 years follow-up, adjusted by breast cancer type (IDC or ILC) (N = 1315).

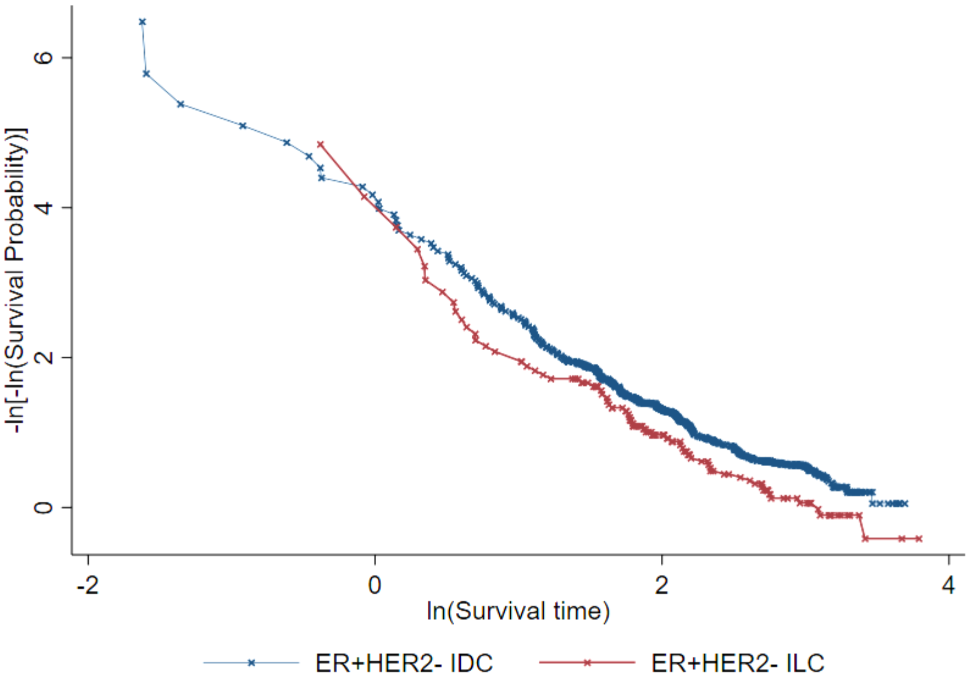

Figure S4. Log-log plot of survival in ER+ HER2- IDC and ER+HER2- ILC (N = 784).

Reference

1. Loibl, S.; Volz, C.; Mau, C.; Blohmer, J.-U.; Costa, S.D.; Eidtmann, H.; Fasching, P.A.; Gerber, B.; Hanusch, C.; Jackisch, C., et al. Response and prognosis after neoadjuvant chemotherapy in 1,051 patients with infiltrating lobular breast carcinoma. *Breast Cancer Research and Treatment* **2014**, *144*, 153-162, doi:10.1007/s10549-014-2861-6.
2. Riba, L.A.; Russell, T.; Alapati, A.; Davis, R.B.; James, T.A. Characterizing Response to Neoadjuvant Chemotherapy in Invasive Lobular Breast Carcinoma. *J Surg Res* **2019**, *233*, 436-443, doi:10.1016/j.jss.2018.08.011.
3. Tamirisa, N.; Williamson, H.V.; Thomas, S.M.; Westbrook, K.E.; Greenup, R.A.; Plichta, J.K.; Rosenberger, L.H.; Hyslop, T.; Hwang, E.-S.S.; Fayanju, O.M. The impact of chemotherapy sequence on survival in node-positive invasive lobular carcinoma. *Journal of surgical oncology* **2019**, *120*, 132-141, doi:10.1002/jso.25492.
4. Cristofanilli, M.; Gonzalez-Angulo, A.; Sneige, N.; Kau, S.W.; Broglio, K.; Theriault, R.L.; Valero, V.; Buzdar, A.U.; Kuerer, H.; Buchholz, T.A., et al. Invasive lobular carcinoma classic type: response to primary chemotherapy and survival outcomes. *J Clin Oncol* **2005**, *23*, 41-48, doi:10.1200/JCO.2005.03.111.
5. Delpech, Y.; Coutant, C.; Hsu, L.; Barranger, E.; Iwamoto, T.; Barcenas, C.H.; Hortobagyi, G.N.; Rouzier, R.; Esteva, F.J.; Pusztai, L. Clinical benefit from neoadjuvant chemotherapy in oestrogen receptor-positive invasive ductal and lobular carcinomas. *Br J Cancer* **2013**, *108*, 285-291, doi:10.1038/bjc.2012.557.
6. Lips, E.H.; Mukhtar, R.A.; Yau, C.; de Ronde, J.J.; Livasy, C.; Carey, L.A.; Loo, C.E.; Vrancken-Peeters, M.-J.T.F.D.; Sonke, G.S.; Berry, D.A., et al. Lobular histology and response to neoadjuvant chemotherapy in invasive breast cancer. *Breast Cancer Research and Treatment* **2012**, *136*, 35-43, doi:10.1007/s10549-012-2233-z.
7. Marmor, S.; Hui, J.Y.C.; Huang, J.L.; Kizy, S.; Beckwith, H.; Blaes, A.H.; Rueth, N.M.; Tuttle, T.M. Relative effectiveness of adjuvant chemotherapy for invasive lobular compared with invasive ductal carcinoma of the breast. *Cancer* **2017**, *123*, 3015-3021, doi:10.1002/cncr.30699.
8. Hu, G.; Hu, G.; Zhang, C.; Lin, X.; Shan, M.; Yu, Y.; Lu, Y.; Niu, R.; Ye, H.; Wang, C., et al. Adjuvant chemotherapy could not bring survival benefit to HR-positive, HER2-negative, pT1b-c/N0-1/M0 invasive lobular carcinoma of the breast: a propensity score matching study based on SEER database. *BMC Cancer* **2020**, *20*, 136, doi:10.1186/s12885-020-6614-0.
9. Truin, W.; Voogd, A.C.; Vreugdenhil, G.; van der Heiden-van der Loo, M.; Siesling, S.; Roumen, R.M. Effect of adjuvant chemotherapy in postmenopausal patients with invasive ductal versus lobular breast cancer. *Ann Oncol* **2012**, *23*, 2859-2865, doi:10.1093/annonc/mds180.
10. de Nonneville, A.; Jauffret, C.; Goncalves, A.; Classe, J.M.; Cohen, M.; Reyal, F.; Mazouni, C.; Chauvet, M.P.; Chopin, N.; Colombo, P.E., et al. Adjuvant chemotherapy in lobular carcinoma of the breast: a clinicopathological score identifies high-risk patient with survival benefit. *Breast Cancer Res Treat* **2019**, *175*, 379-387, doi:10.1007/s10549-019-05160-9.
